# Energy and Quality Evaluation for Compressive Sensing of Fetal Electrocardiogram Signals

**DOI:** 10.3390/s17010009

**Published:** 2016-12-22

**Authors:** Giulia Da Poian, Denis Brandalise, Riccardo Bernardini, Roberto Rinaldo

**Affiliations:** Polytechnic Department of Engineering and Architecture, University of Udine, Via delle Scienze 206, 33100 Udine, Italy; dapoian.giulia@spes.uniud.it (G.D.P.); brandalise.denis@spes.uniud.it (D.B.); bernardini@uniud.it (R.B.)

**Keywords:** fetal ECG, compressive sensing, wearable sensors

## Abstract

This manuscript addresses the problem of non-invasive fetal Electrocardiogram (ECG) signal acquisition with low power/low complexity sensors. A sensor architecture using the Compressive Sensing (CS) paradigm is compared to a standard compression scheme using wavelets in terms of energy consumption vs. reconstruction quality, and, more importantly, vs. performance of fetal heart beat detection in the reconstructed signals. We show in this paper that a CS scheme based on reconstruction with an over-complete dictionary has similar reconstruction quality to one based on wavelet compression. We also consider, as a more important figure of merit, the accuracy of fetal beat detection after reconstruction as a function of the sensor power consumption. Experimental results with an actual implementation in a commercial device show that CS allows significant reduction of energy consumption in the sensor node, and that the detection performance is comparable to that obtained from original signals for compression ratios up to about 75%.

## 1. Introduction

Wearable sensors are a viable and possible solution for continuous monitoring of physiological signals, such as the Electrocardiogram (ECG), during patient’s everyday activities. However, due to the fact that the sensors are often battery operated and have limited computational capabilities, there is increasing interest in the development of low-complexity solutions to acquire, compress and transmit the data, in order to reduce power consumption at the sensor.

Recently, wearable sensors have also been employed for non-invasive abdominal recordings of fetal ECG (fECG) [1,2]. This possibility is useful for remote monitoring of fetus health during the whole pregnancy. The fECG signal, through the manifestation of abnormalities in the morphology of the cardiac electrical signals, allows for identifying possible cardiac defects, making it possible to treat them or to pre-schedule the delivery. It is therefore clear that, for this application, an efficient sensor design and low-complexity compression algorithms in the sensor become particularly important.

In the last few years, Compressive Sensing (CS) has emerged as one of the most promising acquisition/compression paradigms for low-power applications [3,4]. CS exploits the notion of *sparsity*, meaning that a length-*N* signal vector has a small number k≪N of significant coefficients. Under suitable hypotheses, the signal can be reconstructed from a small number *M*∼*k* of measurements, taken as the inner product of the signal with *M* random vectors. Like most natural signals, physiological ones such as the ECG and the fECG signals, are not sparse in themselves, but it is possible to find a sparse representation in an appropriate *sparsifying* basis, e.g., Discrete Cosine Transform (DCT) or wavelets, or an over-complete dictionary, like the one proposed in [5]. Knowledge of the basis or dictionary is required at the receiver for the signal reconstruction procedure.

Energy saving by using CS for *adult* electrocardiogram signal monitoring with wireless sensors has been studied in [6]. A sparsifying wavelet basis is used in the receiver. Authors show that CS is a competitive low-complexity compression paradigm with respect to state-of-the-art Discrete Wavelet Transform (DWT)-based compression. In accordance with the results reported in [6], it is possible to achieve a life extension of the battery up to 37% with an acceptable reconstruction quality. Results are based on an actual implementation on the Shimmer platform (SHIMMER 2R, Shimmer/Realtime Technologies, Dublin, Ireland) [7] for adult ECG signal monitoring, considering a sampling frequency of 256 Hz and 11 bit resolution.

The application of the CS paradigm for *fetal* electrocardiogram signal acquisition, however, introduces some issues due to the nature of the signals acquired using multiple leads, which record a mixture of the fetal, the mother’s heart beats and noise. Indeed, as reported in [8], the use of the traditional wavelet basis is not suitable for the reconstruction of fECG signals from CS measurements. In [8], a new method, namely Block Sparse Bayesian Learning (BSBL), was introduced to overcome the limitations of the traditional CS framework. Exploiting the spatial, temporal and dynamic structure of signals, it enables reconstruction of non-sparse signals with high quality. In particular, the reconstruction process proposed in [8] does not destroy the interdependence structure of multichannel recordings, in order to allow the application of fetal beat detection algorithms, usually based on Independent Component Analysis (ICA) [9] or Blind Source Separation (BSS) [10].

The objective of this work is to explore how effective CS could be for fECG monitoring in battery constrained devices, with limited computational capacity, compared with classical compression techniques, in particular based on DWT. We evaluate the energy consumption performance via actual implementation of the CS and wavelet compression paradigms on the Shimmer platform [7]. This paper proves that, for the analysis of abdominal recordings of fetal ECG signals, which can be difficult to process due to the low-amplitude of the fetal beats, CS, using an appropriate sparsifying dictionary can provide significant advantages with respect to conventional CS schemes based on wavelets, and with respect to DWT schemes in the signal domain. To the authors’ knowledge, this is the first time that such an evaluation has been published. In particular, the contributions of this work are related to three particular aspects. We show that a CS scheme based on reconstruction with an over-complete dictionary, instead of the wavelet basis considered in [8], has similar reconstruction quality to one based on wavelet compression, proving that the CS paradigm is suitable for fECG acquisition, with the advantage of a low power implementation in the sensor. Unlike other works in the literature, we consider, as a figure of merit, the accuracy of fetal beat detection after reconstruction, and compare the results of different compression/transmission/reconstruction procedures as a function of the sensor power consumption. Our results show that the properly designed classical CS paradigm, using an over-complete Gaussian Dictionary at the receiver, can preserve relevant signal information and provide a detection performance comparable to that obtained from original signals for compression ratios up to about 75%.

## 2. Methods

### 2.1. Method Description

**Figure 1:**
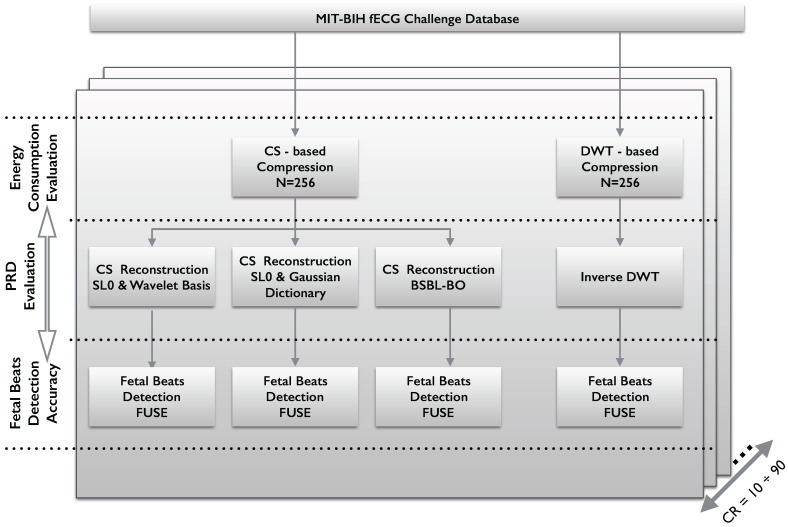
Workflow of the proposed evaluation method.

The workflow adopted for the CS paradigm evaluation in fetal ECG acquisition is reported in Figure 1.

The aim of this work is to compare the energy and reconstruction/detection performance of two encoding procedures, namely CS acquisition (Section 2.2) and DWT compression (Section 2.4).

Energy consumption for the two compression schemes is evaluated at different compression ratios as described in Section 2.6. Both encoding techniques operate on non-overlapping signal blocks of *N* = 256 samples, for signals sampled at 1 kHz with 16-bit resolution.

For the assessment of fetal beat detection accuracy on compressed ECG signals, we consider four different scenarios. The first three scenarios require reconstruction of the ECG signals from compressed sensed measurements. To this end, we adopt two different sparsifying matrices in the decoder reconstruction process. In particular, we use the traditional Daubechies (DB4) wavelet basis with a 5-level decomposition, and the over-complete Gaussian Dictionary proposed in [5,11]. The over-complete dictionary is specifically designed to preserve the relevant waves of both maternal and fetal electrocardiogram signals [11]. The reconstruction algorithm adopted to solve the inverse problem is *λ*SL0 [12], which allows for achieving good performance in the presence of noisy signals and ill-conditioned matrices, while maintaining a relatively low complexity. Moreover, we also consider for comparison the BSBL-Bound Optimization (BSBL-BO) algorithm, used for fECG reconstruction in [8].

In the fourth scenario, the ECG signal is compressed using a DWT-based method (Section 2.4) and, at the decoder, a standard inverse DWT is applied to reconstruct the signals on the basis of the received coefficients.

After signal reconstruction with the two compression schemes (CS or DWT), the FUSE method [13] for fetal ECG extraction and beats detection is applied. The authors have made available the entire FUSE code at [14]. Assessment of the performance of the different scenarios is evaluated using the metrics reported in Section 2.7.

Finally, we combine the energy consumption with the detection performance in order to establish the actual energy saving that one can achieve while guaranteeing a certain detection accuracy.

### 2.2. Data

Since the aim of this work is to comparatively evaluate the CS-based compression schemes and DWT on a standard database, we do not use real-time ECG acquisition to assess the recovery and detection quality. Thus, experiments are carried out using non-invasive fetal ECG signals from set-A of the public database [15]. Due to the inaccuracy of reference annotations, records a38, a46, a52, a54, a71, a74 are discarded, as suggested in [13]. The database contains 75 signals, obtained from multiple sources using a variety of instrumentation with different configurations. The database provides a diversity of fECG recordings, with similar but not identical characteristics. Each signal is one-minute long and includes four non-invasive abdominal signals sampled at 1 kHz, with B=16 bit resolution. For each recording, reference annotations made by experts are available, marking the locations of the fetal QRS complexes, i.e., of the ensemble of the Q, R and S waves of each ECG cycle.

### 2.3. Compressive Sensing Implementation

Compressive Sensing allows for the recovery of sparse or compressible signals from measurements taken at a rate that can be much lower than that required by traditional Nyquist sampling at twice the signal bandwidth [3,4,16].

A signal has a sparse representation if a small number of its coefficients contain a large proportion of the energy. Formally, we say that a signal $x\in R^{N}$ is *k*-sparse when at most *k* of its elements are non-zero, i.e., ${\left||x|\right|}_{0}=\mathrm{Card}\left(\mathrm{Supp}\left(x\right)\right)=k\ll N$. As mentioned, only a few signals are truly sparse in the acquisition domain, but it is generally possible to find a sparse representation x=Ψs, with s sparse, in a transform domain using a *sparsifying* basis or an over-complete dictionary **Ψ**.

Compressive Sensing theory shows that it is possible to reconstruct sparse signals, or, in general, signals $x\in R^{N}$ that have a sparse representation in an appropriate basis or dictionary, from a small number of random projections y=Φx, $y\in R^{M}$, where **Φ** is an M×N matrix whose elements are drawn at random as independent and identically distributed (i.i.d.) random variables from sub-Gaussian distributions, e.g., i.i.d. normalized Gaussian or Bernoulli. Compression derives from the dimensionality reduction obtained by representing the *N*-dimensional signal x with the *M*-dimensional vector y, M<N. The universal encoding sensor has the simple task to compute projections via matrix multiplication, using very low power analog or digital implementations. In real world applications, we deal with nearly sparse signals and measurement noise, and the acquisition model becomes y=Φx+n, where n is an additive term taking errors into account. In such situations, the signal recovery problem, which has to be solved at the receiver, is given by

(1) $$\arg\min_{x}{\left||x|\right|}_{0}\mathrm{subject}\;\mathrm{to}\;||y-{\Phi x||}_{2}\leq \epsilon ,$$

where *ϵ* is a bound on the noise energy , i.e., ${\left||n|\right|}_{2}\leq \epsilon$. Several practical methods have been proposed in order to solve the NP-hard problem in Equation (1) by convex relaxation, such as the basis pursuit denoising (BPDN), where the $l_{0}$ norm in Equation (1), counting the number of non-zero elements in vector x, is replaced by the $l_{1}$ norm and greedy algorithms such as Matching Pursuit (MP) and Orthogonal Matching Pursuit (OMP). In this work, we use the *λ*SL0 algorithm proposed in [12], a modified version of the Smoothed-l0 (SL0) algorithm [17]. The SL0 and *λ*SL0 algorithms solve the problem in Equation (1) by replacing the $l_{0}$ norm with a smooth approximation.

Random sensing matrices, such as Gaussian or Bernoulli matrices, guarantee the recovery of compressed signals with high probability, as shown in [18]. However, when Compressive Sensing is applied in architectures with computational and memory constraints, the use of full random sensing matrices can be problematic due to the relative computational complexity of matrix multiplication in the sensing procedure.

In this work, we use sparse sensing matrices, with two non-zero elements in each column, similarly to the one used in [8]. In particular, the non-zero elements are equal to 1. Thus, the compression stage reduces to the sum of signal samples indexed by the matrix elements that can be implemented using a single accumulator and no floating-point multiplication. Moreover, instead of storing the whole sensing matrix, it is possible to store just the positions of the non-zero elements.

### 2.4. DWT-Based Compression Implementation

**Figure 2:**
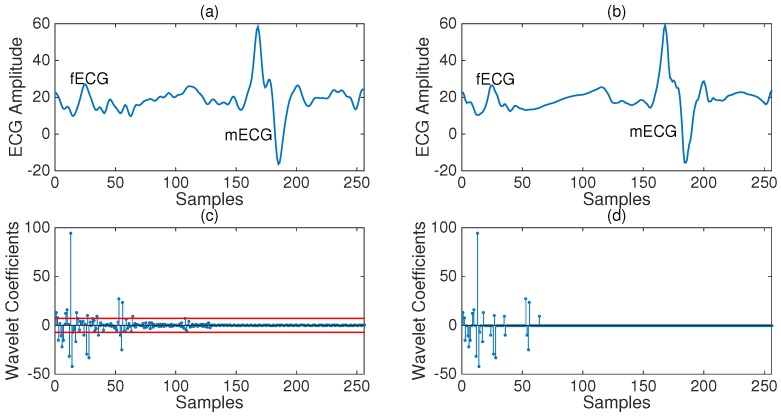
(**a**) original ECG signal and (**c**) corresponding wavelet coefficients, the lines correspond to the threshold level to select the 10% largest coefficients; plots (**b**,**d**) show the reconstructed signal and the 10% largest coefficients used for reconstruction.

DWT [19] allows hierarchical decomposition of an input signal into a series of successively lower frequency approximations and their associated detail signals. As for Compressive Sensing, DWT-based compression is still based on the sparsity principle, since most of the wavelet coefficients of natural smooth signals have a small amplitude, so that the signal is approximately sparse in the wavelet domain. Indeed, the smallest wavelet coefficients can be neglected without much signal quality loss, as shown in Figure 2 where only 10% of the original coefficients are kept. Thanks to this property, the wavelet transform is widely used for the compression of signals and images.

The DWT-based compression scheme needs to compute the transform in the sensor, and then exploit sparsity by transmitting a subset of the computed coefficients. The DWT is computed with a cascade of filters followed by a factor 2 subsampling. The resulting low-pass coefficients represent a rough subsampled approximation of the original signal, while the high-pass coefficients represent detailed information. Due to subsampling, the number of wavelet coefficients is *N* for a length-*N* input signal vector (when using appropriate extensions of the signals at the borders.)

In this work, we use the orthogonal Daubechies wavelet (DB4) with 8-tap filters, which provides a sparse representation for piecewise-linear signals and thus is suitable for ECG signals, leading to a relatively sparse representation with most of the coefficients close to zero (see Figure 2).

Our choice considers a 4-level wavelet decomposition and 256 sample frames. Before applying the DWT, the ECG signal block is preprocessed to remove the mean value. In order to have accurate power consumption estimates in a concrete scenario [6], we implemented the algorithms on a general-purpose MSP430 [20] microcontroller that does not include a floating-point unit. Therefore, all computations are performed in fixed-point, which is suitable for real-time embedded applications. Filter coefficients are represented with nine bits, and the original abdominal ECG samples in x and the corresponding wavelet coefficients $\alpha =\left[d1,d1,d3,d4,a4\right]$ are both represented with B=16 bits.

Signal compression is performed by keeping the largest DWT coefficients, as suggested in [21]. In this work, the number of retained coefficients is selected on the basis of the desired compression ratio. In particular, given a compression ratio and the relative number of coefficients to retain $N_{coeff}$, only the first largest $N_{coeff}$ coefficients in absolute value are kept. The largest coefficients are selected using the ordering algorithm *merge sort*, which has a computational complexity O($N\log_{2}N$).

In summary, two vectors are used to code the DWT coefficients. One contains only the non-zero wavelet coefficients, and the other one contains the corresponding positions. Both need to be sent to the receiver in order to recover the signal.

### 2.5. Fetal Beat Extraction and Detection

Several methods for fetal beat detection from abdominal ECG recordings have been proposed in the last few years [22,23]. Here, we consider methods for fetal beat detection in multi-channel non-invasive abdominal ECG recordings, without maternal ECG reference. Usually, the proposed methods consist of a pre-processing stage, whose objective is to remove baseline wandering and power-line interference. The second step consists in the estimation of maternal beats by using a decomposition technique, like Independent Component Analysis (ICA) or Singular Value Decomposition (SVD). The third stage consists of removing the maternal component through sub-space reconstruction, maternal template subtraction or filtering. Finally, after the maternal component has been removed, the fourth step consists of the detection of fetal QRS complexes. After this final step, it is possible to further post-process the detected beats to constrain the estimated Fetal Heart Rate (FHR) and RR time series within physiological or statistical limits based on heuristics. RR represents the time interval between two consecutive R waves.

In this work, we use the FUSE method proposed in [13], which is a combination of template subtraction and principal/independent component analysis. The final fetal QRS complex detection is performed by using a Pan and Tompkins QRS detector on all of the channels [24]. Then, the one with the smoothest fetal heart rate time series is selected.

### 2.6. Energy Consumption Evaluation

Energy requirements of the two different compression algorithms are evaluated on the basis of the actual number of microcontroller unit (MCU) cycles and transmission bitrate required by implementation in a commercial acquisition device. Since energy consumption does not depend on the actual signals, we evaluate the cost of the algorithms in a real implementation, although experiments, for comparison purposes, are carried out off-line on signals of the public database described above.

The hardware considered in this work is the one present on Shimmer devices [7] powered by a rechargeable Li-polymer battery, with an internal ECG daughter board, validated for ambulatory and research purposes. In our experiments, the sampling rate of the device is set to 1 kHz. The device includes the low-power Texas Instruments 16-bit MSP430F5438 micro-controller (Texas Instruments Inc., Dallas, TX, USA) [20], and a low-power CC2420 IEEE 802.15.4 (Texas Instruments Inc., Dallas, TX, USA) [25] The MSP430 is a 16-bit word processor, and the compression performance of both algorithms is evaluated using 16 bit arithmetic. Code Composer Studio (CCS) (version 6.0, Texas Instruments Inc., Dallas, TX, USA) has been used to generate the firmware binary code. One of the functionalities of the CCS development kit allows for counting MCU cycles for the running code.

We consider the energy cost E${}_{comp}$ required by the compression algorithms to process one signal block of N=256 samples. E${}_{comp}$ can be expressed as $E_{comp}=N_{cyc}E_{cyc},$ where $N_{cyc}$ is the number of MCU cycles to encode one signal block.

The energy consumption per clock cycle E${}_{cyc}$ can be easily calculated for the considered micro-controller, which, in active mode, consumes 312 μA/MHz when the MCU operates at 8 MHz and the supply voltage is +3 V, namely

(2) $$E_{cyc}\left(@8\mathrm{MHz}\right)=312\cdot 3\cdot 10^{-12}=0.936\mathrm{nJ}/\mathrm{cycle}.$$

Since the two compression schemes may require different bitrates for the same reconstruction quality or beat detection capability, we also consider, in the following, the transmission cost, which usually has the highest impact on the overall energy consumption. To this purpose, we take into account the CC2420 radio specifications (Texas Instruments Inc., Dallas, TX, USA) [25]. In [25], it is reported that the energy consumption per transmitted bit is E${}_{bit}$ = 230 nJ/bit.

The transmission energy, E${}_{tx}$, to transmit one signal block equals therefore

(3) $$E_{tx}=N_{bit}E_{bit},$$

where $N_{bit}$ is the number of bits necessary to encode the block.

Finally, the total energy required to process and transmit a signal block is given by.

(4) $$E_{TOT}=E_{comp}+E_{tx}=N_{cyc}E_{cyc}+N_{bit}E_{bit}.$$

In the following, we do not consider the energy required by the Analog to Digital Converter (ADC), since the cost is the same in both scenarios.

### 2.7. Reconstruction Quality Assessment

In order to assess the quality of the reconstructed signals, we use the traditional Percentage Root-mean-square Difference (PRD) quality metric, defined as

(5) $$\mathrm{PRD}\left(\%\right)=\sqrt{\frac{\sum_{n}{\left(x\left(n\right)-\hat{x}\left(n\right)\right)}^{2}}{\sum_{n}x{\left(n\right)}^{2}}}\times 100,$$

where x(n) and $\hat{x}\left(n\right)$ are the original and reconstructed signals, respectively. In evaluating the PRD measure, it is assumed that both signals are zero-mean. In the experiments described below, the PRD value is computed for each reconstructed signal block in every channel and then the average and standard deviation are reported. According to [26], reconstructions with PRD values between 0% and 2% are qualified to have “very good” quality, while values between 2% and 9% are categorized as “good”.

Moreover, the results of fetal beat detection are used as a reconstruction quality measure. In particular, we evaluate the Sensitivity (S) and the Positive Predictivity (P+), based on the comparison between the reference markers and the detected beats for each database signal, which consists of 4-channel recordings. According to the American National Standard [27], S and P+ are computed as

(6) $$S=\frac{\mathrm{TP}}{\mathrm{TP}+\mathrm{FN}}100,\;\;\;\;P+=\frac{\mathrm{TP}}{\mathrm{TP}+\mathrm{FP}}100,$$

where TP is the number of true positives, FP of false positives and FN of false negatives. A detected beat is considered to be true positive if its time location differs less than 50 ms from the reference markers (within a window of 100 ms centered on the reference marker). In the following, we report average S and P+ values for the database signals, together with the standard deviation.

## 3. Experimental Results

### 3.1. Energy Consumption in the Sensor

**Figure 3:**
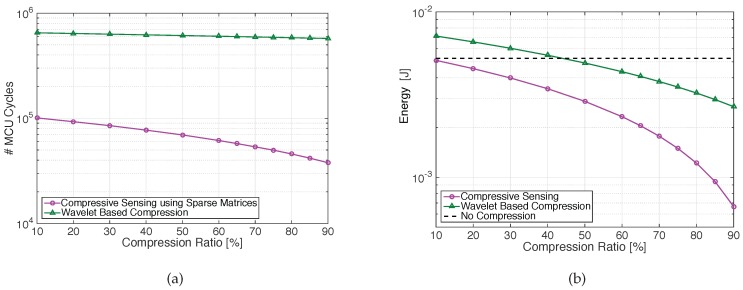
( **a**) number of MCU cycles required to compress a signal block (*N* = 256 samples) using sparse random matrices or DWT as a function of the compression factor; and (**b**) energy required to compress and transmit one N=256 signal block for each channel in a 4-channel recording (four blocks in total), using the CS or DWT-based schemes.

In Figure 3a, the number of cycles required by the microcontroller to perform the compression of a single N=256 signal block for the two coding schemes is shown. The computational workload required for the wavelet scheme, which includes filtering and multiplications, is significantly higher than that required by the CS scheme, requiring additions only. As an example, to compress at CR = 50% 1 s of one channel of ECG data, the wavelet based compression code executes in about 306 ms, whereas the CS code only requires about 35 ms. Note that DWT does not allow real-time processing for 4-channel recordings sampled at 1 kHz, and would require buffering at the sensor or using a smaller sampling frequency. The required total energy, including the one for transmission, is shown in Figure 3b for the two algorithms at various compression ratios. In particular, results refer to the energy required for the compression and transmission of four blocks of length *N* = 256, corresponding to 250 ms in 4-channel recording.

### 3.2. Signal Quality and Detection Performance

**Figure 4:**
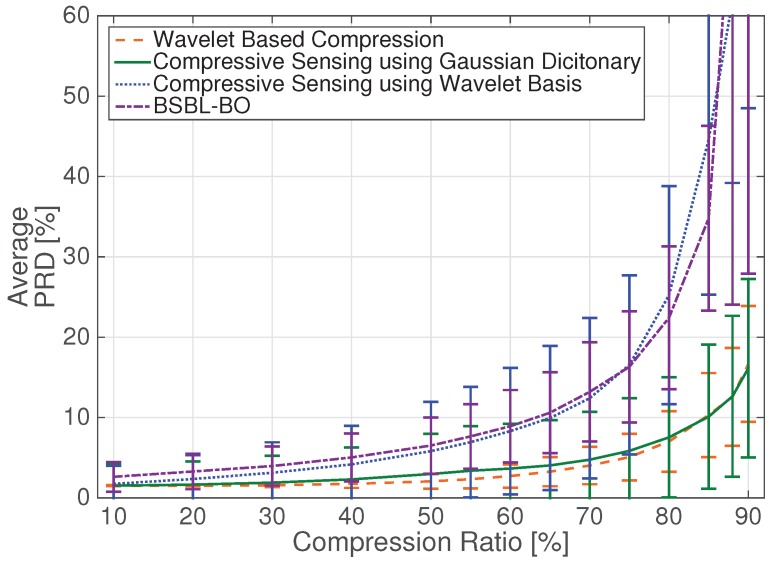
Average PRD values for different compression/ reconstruction schemes. Error bars indicate standard deviation.

**Figure 5:**
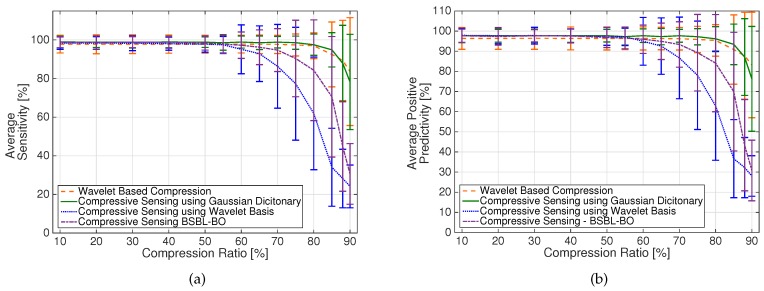
(**a**) average Sensitivity values and (**b**) average Positive Predicitivity values for different compression/reconstruction schemes. Error bars indicate standard deviation.

In Figure 4 and Figure 5, we report the average PRD value, for reconstruction quality assessment, and the average Sensitivity and Positive Predictivity, in order to verify the accuracy of the detection algorithms resulting from the CS and DWT-based schemes.

To compare how well the different techniques can preserve relevant signal characteristics, Figure 5a,b show the average Sensitivity and Positive predicitivity measures obtained from the application of the detection algorithm on the reconstructed signals.

**Figure 6:**
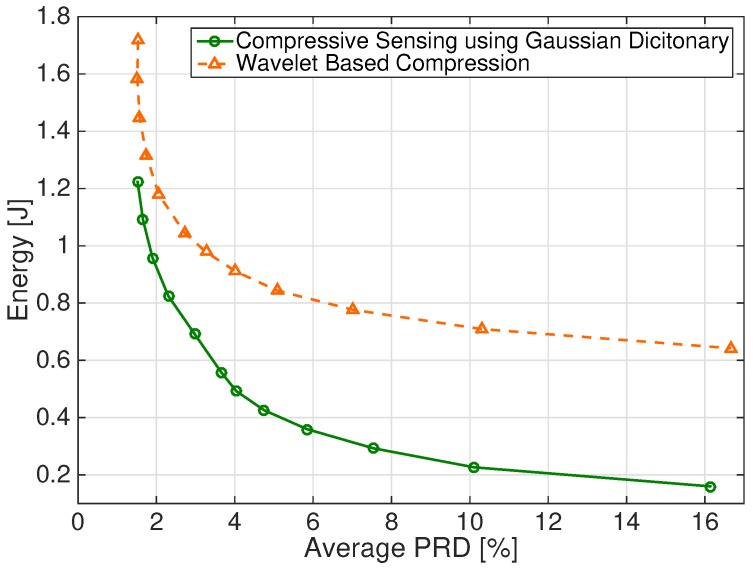
Energy required by the DWT-based and CS schemes to achieve a desired PRD value. Energy values refer to a 4-channel, 1 min long signal.

Figure 6 shows the energy required by the two schemes as a function of the PRD value. In particular, we report the energy values necessary to compress and transmit the entire 4-channel, 1 min long signals.

**Figure 7:**
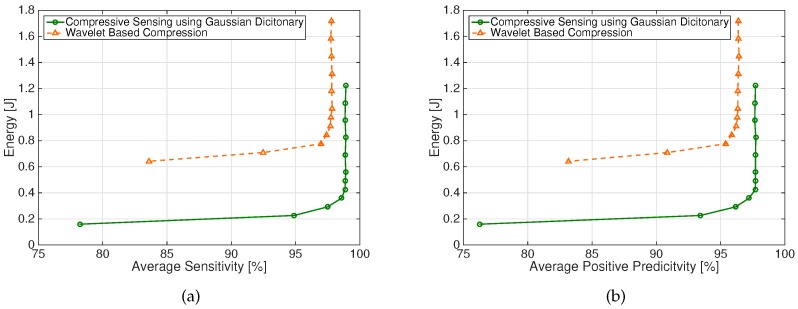
Energy required by the DWT-based and CS schemes to achieve a desired (**a**) average Sensitivity value and (**b**) average Positive Predicitivity value. Energy values refer to a 4-channel, 1 min long signal.

The energy required to achieve a desired fetal beat detection Sensitivity and Positive Predictivity is reported in Figure 7a,b, respectively.

## 4. Discussion

Extraction of fetal ECG signal from abdominal non-invasive recording is a challenging task even on raw uncompressed data. In our study, we investigated the effect of CS-based ECG as well as DWT-based compression on the accuracy of a state-of-the-art fetal beats detector, for a wide range of compression ratios between 10% and 90%. In particular, we assessed the energy required by the two compression schemes.

In terms of energy, results in Figure 3b show that compressive sensing is more energy-efficient than transmitting the uncompressed original signal (for one signal block, $E_{comp}=0,E_{tx}=NBE_{bit}$) for compression ratios CR > 10%, while the DWT-based compression becomes favorable for CR > 45%. Figure 3a,b also show that when increasing the compression ratio, the gap between the two compression techniques increases, leading to higher energy-saving for the CS-based method. It is clear that CS allows for reducing the encoding complexity in the sensor node, allowing a reduction of the overall energy consumption.

The experiments summarized in Figure 4, Figure 5, Figure 6 and Figure 7, assess how the different compression schemes impact the reconstruction and detection quality of the signal at the receiver. In particular, for the CS-based scheme, the different methods described above are used for reconstruction.

As it can be seen from Figure 4, the DWT-based scheme allows for having a good reconstruction quality up to CR = 80%. In previous works, e.g., [6], the performance of DWT-based compression was compared to a CS scheme where the optimization reconstruction problem was solved at the receiver using a wavelet basis as the sparsifying matrix **Ψ**, showing that the quality of the recovered signal was in favour of the DWT-based scheme. Figure 4 confirms that CS reconstruction using the wavelet basis at the receiver (dotted line) has lower performance, i.e., higher PRD values, than DWT-based compression (dashed line). Using the specifically designed dictionary [11], however, the performance of the DWT-based scheme and CS scheme (continuous line in Figure 4) become similar in terms of average PRD value. Indeed, both algorithms allow compression up to CR = 80% maintaining a good reconstruction quality. The CS-based approach, however, requires significantly lower energy, as discussed below. Figure 4 also shows PRD values for the BSBL reconstruction technique in the CS scenario (dashed-dotted line). The performance is similar to the one obtained using CS and the wavelet sparsifying basis at the receiver. However, we will confirm below that BSBL better preserves signal characteristics and allows for improved detection performance after signal reconstruction.

The average Sensitivity and Positive Predictivity measures in Figure 5a,b show that the different techniques have approximately the same performance for compression ratios less than 50%, with S and P+ values very similar to those obtained from the uncompressed signals, i.e., S = 98.9% and P+ = 97.7%. At higher compression values, the DWT-based and CS/Gaussian dictionary methods give considerably better results. Note that the BSBL reconstruction technique (dashed-dotted line) outperforms CS reconstruction with the wavelet basis at the receiver (dotted line), confirming that BSBL can preserve dependency among ECG channels, which is exploited by ICA in the detection algorithm. However, performance achieved adopting the BSBL method is still lower than that obtained with the DWT-based scheme (dashed line) and CS with Gaussian dictionary reconstruction at the receiver (continuous line). For these techniques, the S and P+ values are approximately constant up to CR = 75%, with values similar to those obtained with uncompressed signals.

It is apparent from Figure 6 and Figure 7a,b that the considered CS scheme allows significant energy saving for all the considered figures of merit. For instance, for PRD = 9%, less than 0.3 J are required by the CS scheme, while the DWT-based scheme requires about 0.7 J. A sensitivity value S = 95% requires about 0.2 J and 0.7 J for DWT-based and CS schemes, respectively. Similar values are required to have P+ = 95% for the two schemes.

**Figure 8:**
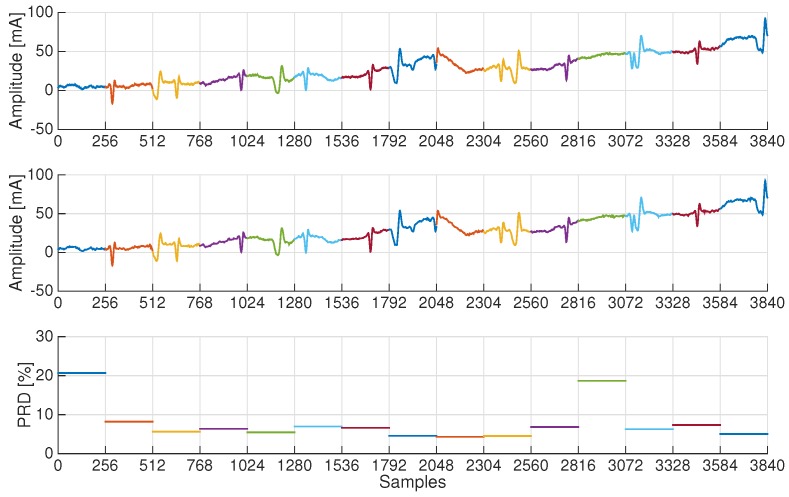
(**top**) original and (**middle**) reconstructed record a28 after CS compression at CR = 70% using the Gaussian dictionary for sparsification; (**bottom**) corresponding PRD value for each window.

As a matter of fact, PRD values can provide, in some cases, a rough estimate of the signal quality, especially when the interest is preserving clinically relevant aspects. As an example, we report in Figure 8 one ECG trace and the corresponding PRD values for each signal block. It can be noticed that blocks with the largest PRD values do not contain relevant ECG information. Clearly, a noisy block cannot be sparse in the wavelet basis or Gaussian Dictionary representations, thus worsening the performance of CS-based schemes.

In summary, the DWT-based scheme and the CS scheme with Gaussian dictionary at the receiver appear to have comparable performance in terms of PRD, S and P+ metrics. However, the CS scheme allows significant energy-saving in the encoding sensor.

## 5. Conclusions

In this paper, we evaluated energy consumption of two acquisition schemes for multi-channel abdominal fECG signals, one based on DWT and the other based on the emerging CS paradigm. Experimental results with an actual implementation on a commercial device show that compressive sensing allows for significantly reducing energy consumption in the sensor node. Moreover, it is advantageous with respect to transmission of the uncompressed signals for compression ratios higher than 10% (the DWT-based scheme becomes preferable only for CR > 45%). We compared the quality of the recovered signal in terms of PRD values, and also, more importantly, by testing the performance of a state-of-the-art fetal beat detector on the recovered traces. We found that compressive sensing, using a suitable dictionary for signal sparsification at the receiver, can achieve the same results of the DWT-based scheme, but with significantly lower energy consumption. In particular, we showed that the detection performance obtained with the CS scheme is comparable to that obtained from original signals for compression ratios up to about 75%. This study confirms that compressive sensing, by moving complexity to the receiving end, where the reconstruction optimization algorithm, together with other processing tasks, is run, is indeed suitable for fECG monitoring in low power applications, and allows the use of sensors with limited complexity.
